# Case report: Surgical repair of a large tracheo-esophageal fistula in a patient with post-transplant esophageal lymphoproliferative disorder

**DOI:** 10.1016/j.ijscr.2022.107537

**Published:** 2022-08-24

**Authors:** Jonathan Schumacher, Christian Alexander Gutschow, Ilhan Inci, Viktor H. Koelzer, Isabelle Opitz

**Affiliations:** a Department of Thoracic Surgery, University Hospital Zurich, Zurich, Switzerland; b Department of Surgery and Transplantation, University Hospital Zurich, Zurich, Switzerland; c Department of Pathology and Molecular Pathology, University Hospital Zurich, Zurich, Switzerland

**Keywords:** Malignant tracheo-esophageal fistula, Post-transplant esophageal lymphoproliferative disorder, Subtotal esophagectomy, Case report

## Abstract

**Introduction and importance:**

The management of large malignant tracheo-esophageal fistulas (TEF) is not standardized. Herein, we report a case with a malignant TEF associated with esophageal post-transplant lymphoproliferative disorder (PTLD) for whom we successfully performed a surgical repair. This contributes to the knowledge on how to treat large acquired malignant TEFs.

**Case presentation:**

A 69-year old male presented with a one-week history of fever, productive cough and bilateral coarse crackles. In addition, he described a weight loss of 10 kg during the past three months. The patient's history included a kidney transplantation twenty years ago. Esophagogastroduodenoscopy with a biopsy of the esophagus was performed nine days before. Histopathology showed a PTLD of diffuse large B-cell lymphoma subtype. Subsequent diagnostics revealed a progressive TEF (approx. 2.0 × 1.5 cm) 3.0 cm above the carina. PET-CT scan showed an esophagus with slight tracer uptake in the middle third (approx. 11.5 cm length, SUV max 7.4). After decision against stenting, transthoracic subtotal esophagectomy with closure of the tracheal mouth of the fistula by a pedicled flap was performed. PTLD was treated with prednisone and rituximab. Tumor progression (brain metastasis) led to death 95 days after surgery.

**Clinical discussion:**

The treatment of a malignant TEF is complex and personalized while both the consequences of the esophago-tracheal connection and those of the underlying responsible diagnosis have to be considered concurrently. In this case, we considered surgery as the best treatment option due to a relatively good prognosis of the underlying diagnosis (PTLD) and a large fistula. Esophageal or dual stenting, the treatment of choice for small malignant TEF, would have been associated with a high risk of failure due to the wide trachea, extensively dilated esophagus, proximal location and large diameter of the fistula.

**Conclusion:**

Surgery can be considered for patients with a large acquired malignant TEF and positive long-term prognosis of the underlying diagnosis. Due to the complexity of TEF management, immediate pre-operative multidisciplinary discussion is advised.

## Highlights

• Surgical repair of a large malignant tracheo-esophageal fistula
• Immediate pre-operative multidisciplinary discussion is needed for large malignant trachea-esophageal fistulas.
• Esophageal post-transplant lymphoproliferative disorder (PTLD) of diffuse large B-cell lymphoma (DLBCL) subtype
• Esophagectomy and closure of the trachea by a pedicled deepithelized muscle flap

## 1 Introduction

Tracheo-esophageal fistula (TEF) is a pathological connection between the trachea and the esophagus, which can be categorized mainly into congenital and acquired cases. Acquired TEF is divided into benign and malignant categories, whereas the latter accounts for more than 50 % of the acquired cases [1]. While benign TEF occurs e.g. after prolonged mechanical ventilation, blunt chest or neck trauma or ingestion of foreign bodies, malignant TEF mostly occurs in patients with esophageal (79 %), tracheal, laryngeal, or primary lung cancer [1]. On average, untreated acquired malignant TEF leads to death within 1 to 6 weeks, often due to aspiration pneumonia [1]. While the management of congenital and benign TEF is well described, standardized treatment recommendations for malignant TEF are lacking. Small malignant TEFs are mostly managed by esophageal and/or tracheal stenting for the prevention of dysphagia and aspiration respectively, sometimes combined with radiation therapy. The management of patients with large malignant TEFs varies to a great extent, due to the patients' poor general condition, malnutrition, and pulmonary compromise [2]. Surgery as a curative approach is often only applicable for transient, well-nourished benign cases, because malignancy is generally associated with a high morbidity, mortality, and recurrence rate, depending on tumor histology [1], [3], [4].

In this case, we successfully managed a large malignant TEF by performing a transthoracic subtotal esophagectomy and a closure of the defect in the membranous part of the trachea with a pedicled deepithelized latissimus dorsi flap.

This work has been reported in line with the SCARE 2020 criteria [5].

## 2 Presentation of case

A 56-year old male patient presented with a one-week history of fever, chills, productive cough, and bilateral coarse crackles. Furthermore, he described a weight loss of 10 kg during the past three months. The patient's history included a kidney transplantation twenty years ago due to a diabetes mellitus type 1-induced nephropathy. Since then, immunosuppression lead to at least three periods of polymicrobial esophageal thrush, including *Candida albicans*, cytomegalovirus (CMV) infection, and others.

Nine days before presentation, elective esophagogastroduodenoscopy with a biopsy of the esophagus was performed. Histopathology showed polymicrobial esophageal thrush (*Serratia marcescens*, *Lacticaseibacillus rhamnosus*, *Streptococcus mittis*, *Streptococcus anginosus*, *Candida albicans*, CMV) and, additionally, a monomorphic post-transplant lymphoproliferative disorder (PTLD) of diffuse large B-cell lymphoma (DLBCL) subtype. CMV viremia was confirmed by polymerase chain reaction, which in the large majority of cases corresponds to a reactivation of latent CMV infection.

**Fig. 1:**
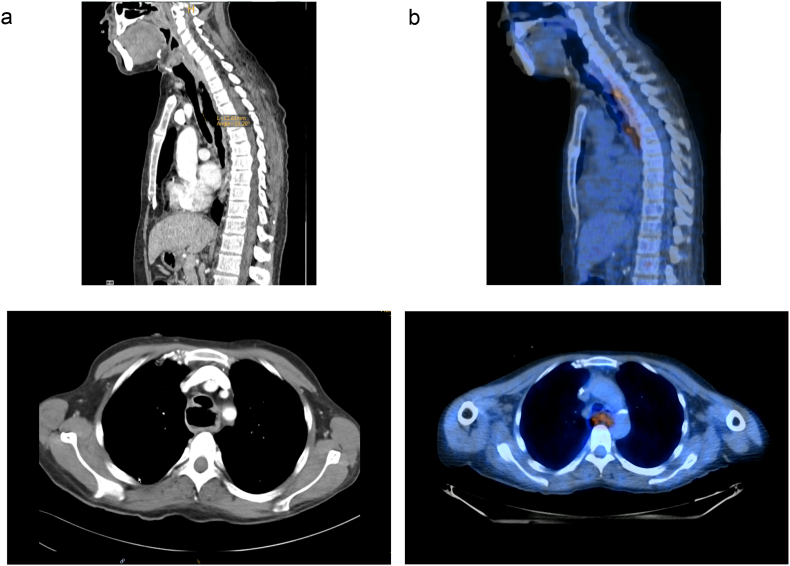
Initial imaging. a) CT-scan sagittal (top) transversal (bottom) showing extensively dilated and wall-thickened esophagus (42 × 23 mm), a tracheo-esophageal fistula (6 × 13 mm) on the level of the T3 vertebral body. b) PET CT scan sagittal (top) transversal (bottom) showing a slightly activated 11.5 cm long esophagus (middle third, SUV max 7.4) without locoregional lymphadenopathy.

Initial neck and thoracic contrast enhanced computer tomography (CT) scan revealed an extensively dilated and wall-thickened esophagus (42 × 23 mm), a TEF (6 × 13 mm) on the level of the T3 vertebral body, and signs of aspiration pneumonia (Fig. 1a). PET-CT scan showed an esophagus with slight tracer uptake in the middle third (approx. 11.5 cm length, SUV max 7.4) without locoregional lymphadenopathy (Fig. 1b). Head MRI showed three small ring-shaped enhancing lesions in occipital, frontal and parietal lobe, morphologically highly suspicious for multifocal tumor lesions in the brain. Bone marrow puncture was not performed. Cerebrospinal fluid ruled out an infection with *Toxoplasma gondii*, *Mycobacterium tuberculosis*, or *Cryptococcus* species. Therefore, brain metastasis was assumed.

**Fig. 2:**
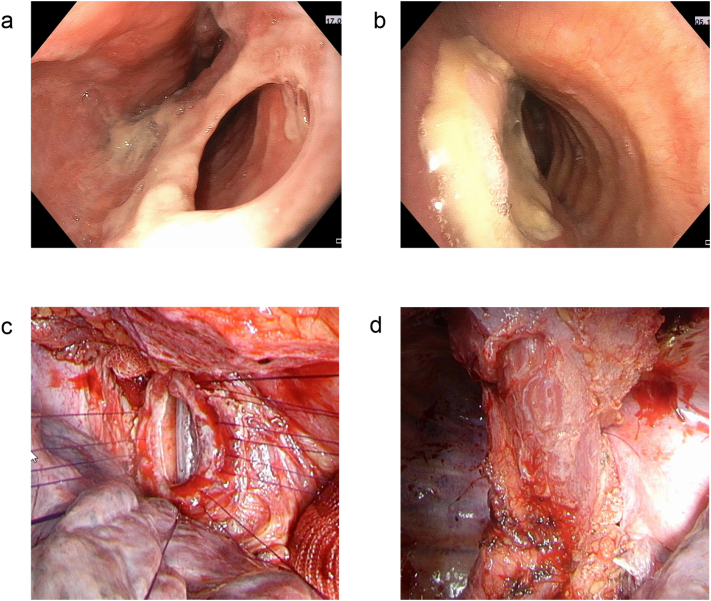
a) Gastroscopy confirming a large communicating tracheo-esophageal fistula in the membranous part of the trachea approx. 2.0 cm in craniocaudal direction and 3.0 cm above the carina. b) Follow up bronchoscopy 12 days after surgery showing a sufficient closure with the pedicled flap. c) Intraoperative picture showing the trachea before closure. d) Intraoperative picture showing the trachea after closure with a pedicled latissimus dorsi flap.

Six days later, after transfer to our institution, gastro-/bronchoscopy confirmed a large communicating TEF in the membranous part of the trachea approx. 2.0 cm in craniocaudal direction and 3.0 cm above the carina (Fig. 2a). After interdisciplinary discussion, a decision against stenting was made due to its high chance of failure in this case (wide trachea, extensively dilated esophagus, proximal location and large diameter of the fistula). Preoperative temporary esophageal stenting was necessary. A transthoracic subtotal esophagectomy with indirect closure of the tracheal mouth of the fistula by a pedicled deepithelized myocutaneous latissimus dorsi flap was performed (Fig. 2c and d). Furthermore, cervical esophagostomy and laparoscopic implantation of a percutaneous endoscopic gastrostomy completed initial surgical treatment and gastric pull-up was planned in a second step. Experts in thoracic and upper gastrointestinal surgery performed the surgery. Follow-up bronchoscopy showed a sufficient closure with pedicled flap (Fig. 2b). Post-operative complications were a hypoxemic respiratory failure due to hypervolemia, an anuric acute kidney injury (both successfully treated by hemofiltration), a conservatively treated paralytic ileus, and a unilateral vocal fold paralysis (Clavien-Dindo Classification Grade IVa due to hemofiltration [6]). Due to the TEF it was assumed that the polymicrobial spectrum of the esophageal thrush caused aspiration pneumonia. Aspiration pneumonia and CMV infection were controlled by antibiotics (cefepime and metronidazole), fluconazole and ganciclovir. PTLD was treated with prednisone and rituximab for 30 days, further, azathioprine was stopped.

**Fig. 3:**
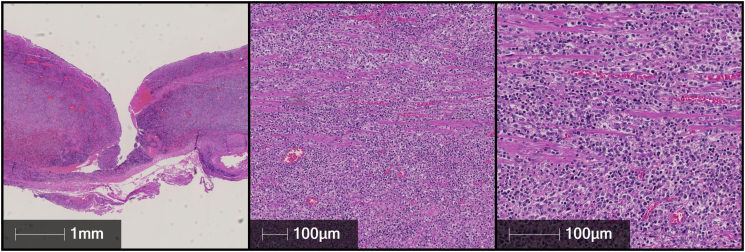
Histological images (Hematoxylin Eosin stain (HE), 2×, 10×, 20×) of the esophagectomy specimen showing a diffuse infiltration of the esophageal wall by a monomorphic lymphoid infiltrate in the region of transmural perforation (fistula).

**Fig. 4:**
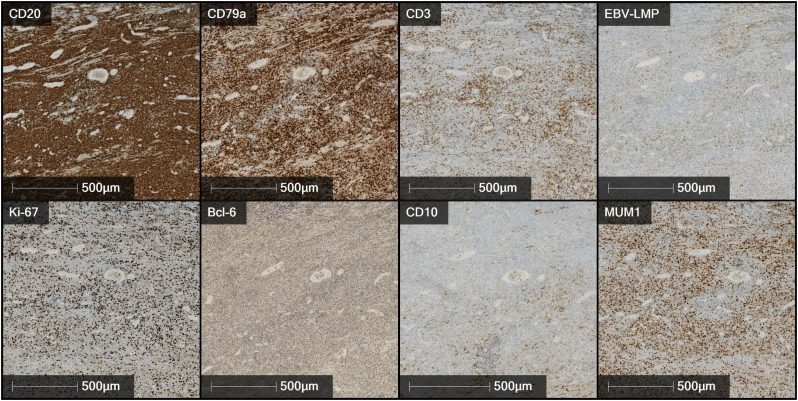
Immunohistochemical profile of the neoplastic cell population: Reactivity of the neoplastic cells for CD20, CD79a and IRF4/MUM1 (CD19 and PAX-5 positive, not shown). CD3 marks a diffuse, concomitant T-cell infiltrate. Weak, partial reactivity of the neoplastic cell elements for bcl-6, no reactivity for CD10 and CD30. Reactivity for EBV latent membrane protein (LMP) in approximately 20 % of the neoplastic cell population. Proliferation fraction (ki-67) of approximately 40 %. The immunohistochemical expression profile is consistent with the diagnosis of a diagnosis of a monomorphic post-transplantation lymphoproliferative disease (PTLD) of the diffuse, large B-cell lymphoma type, EBV-associated.

Pathological examination of the esophagectomy specimen revealed transmural infiltrates of a monomorphic, mass-forming lymphoid infiltrate with circumscribed perforation of the esophageal wall (Fig. 3). Immunohistochemical examination confirmed the diagnosis of a monomorphic post-transplantation lymphoproliferative disease (PTLD) of the diffuse, large B-cell lymphoma type, EBV-associated (Fig. 4).

Two and a half months after surgery, head MRI – performed due to persistent headache – revealed multiple large necrotic lesions in the cerebrum. Due to polymorbidity, a decision against combination chemotherapy with curative intent was made. Due to missing consequences, brain biopsy was not performed and progress of PTLD was assumed; there was no clinical or radiographic evidence of a secondary neoplasia. After whole-brain radiotherapy and treatment with dexamethasone and rituximab, tumor progression led to death 95 days after surgery.

During a follow-up six weeks after surgery, the patient reported an improved lung function and mobility; however, he still suffered from a quick fatigue and lost an additional four kilograms of weight. Since the gastric pull up could not be performed due to tumor progression, a final detailed assessment of the patient and his perspective on the procedure was not done.

Length of hospital stay was 56 days (preoperative: 12 days; postoperative: 43 days, including 12 days in our center and 31 days in a regional tertiary referral hospital), followed by 19 days in a rehabilitation center. Before the patient was discharged to a palliative care home, the patient stayed another 22 days in a regional tertiary hospital due to hyperglycemia and re-staging (head MRI).

## 3 Discussion

In this case, we considered surgery as the best treatment option due to a relatively good prognosis of the underlying diagnosis and a large fistula with high risk of stent failure.

The patient suffered from an extended esophageal monomorphic PTLD of diffuse large B-cell lymphoma (DLBCL) subtype which is an exceedingly rare diagnosis. In a multicenter, international analysis approximately 40 % of patients with primary central nervous system PTLD showed a three-year overall survival after therapy with rituximab and/or combination chemotherapy [7]. Thus, compared to the common underlying malignancies in TEF, such as primary esophageal or laryngeal cancer, the patient was considered to have a better long-term prognosis.

In addition, esophageal or dual stenting, the treatment of choice for small malignant TEF, would have been associated with a high risk of failure due to the wide trachea, extensively dilated esophagus, proximal location and large diameter of the fistula without great potential of spontaneous closure [8]. Stenting (in particular dual stenting due to the opposing radial forces), radiation- and chemotherapy tend to induce tumor necrosis, shrink the tumor and therefore accelerate the fistula [3], [4], [9]. Other options beside stenting include cervical esophagostomy with gastro- or jejunostomy only, or an esophageal bypass. In general, using surgical methods for malignant TEF has either been declared impossible to carry out or has been associated with a high morbidity and mortality [1], [10], [11]. However, due to the extended PTLD manifestation with a relatively good prognosis in combination with a large TEF, transthoracic subtotal esophagectomy, cervical esophagostomy, and implantation of a percutaneous endoscopic gastrostomy was performed. Gastric pull-up was planned in a second step, depending on the patients' postoperative course.

The tracheal mouth of the fistula was indirectly closed by a pedicled deepithelized myocutaneous flap of latissimus dorsi muscle. A pedicled flap is connected to its original blood supply, thereby avoiding the insufficient healing and early recurrence associated with former ways of patching, using fascia, skin and other foreign materials. Moreover, to limit further spread of the malignancy, therapy with prednisone and rituximab was initiated. Other approaches would have been to resect or directly suture the tracheal part of the fistula. Direct suture is not an option in large malignant fistulas due to airway stenosis. Nowadays, planned resections of up to 4 cm of the trachea will mostly be tolerated, depending on the surgical approach (cervical or transthoracic) and individual conditions (age, posture, body habitus, extent of disease, prior tracheal surgery) [12]. However, we decided against a technically challenging tracheal resection and reconstruction in the light of our patients' immunodeficiency (transplantation, prednisone and rituximab for PTLD, diabetes mellitus type 1, chronic obstructive pulmonary disease), the large diameter of the fistula and its proximity to the tracheal bifurcation and concomitant esophageal major surgery.

Overall survival after surgery in this patient was 95 days. The patient spent all of the time in a hospital (9 weeks) or in other institutions (rehabilitation center, palliative care home).

In general, since both the consequences of the esophago-tracheal connection and those of the underlying responsible diagnosis have to be considered concurrently, treatment of an acquired malignant TEF is complex and personalized. Therefore, the individual approach should ideally be discussed within a multidisciplinary team including thoracic and upper gastrointestinal surgeons, interventional pulmonologists, gastroenterologists, oncologists, and anesthesiologists.

## 4 Conclusion

Surgery can be considered for patients with a large acquired malignant TEF, stable physical condition, and relatively positive long-term prognosis regarding tumor histology and stage. Nonetheless, immediate pre-operative multidisciplinary discussion is needed.

## Consent

Written informed consent was obtained from the daughter of the patient for publication of this case report and accompanying images. A copy of the written consent is available for review by the Editor-in-Chief of this journal on request.

## Provenance and peer review

Not commissioned, externally peer reviewed.

## Ethical approval

In Switzerland, case reports are not required to be voted by the Swiss Ethics. If required, this specific confirmation can be given later.

## Funding

None declared.

## Guarantor

Isabelle Opitz.

## Research registration number

N/a.

## CRediT authorship contribution statement

1. Conceptualization: JS, CAG, II, IO.
2. Data curation: JS, CAG, II, VHK, IO.
3. Formal analysis: JS, CAG, II, VHK, IO.
4. Funding acquisition: n/a.
5. Investigation & Methodology: JS, CAG, II, VHK, IO.
6. Project administration: IO.
7. Resources: JS, CAG, II, IO.
8. Software: n/a.
9. Supervision: IO.
10. Validation: JS, II, IO.
11. Visualization: JS, II, VHK, IO.
12. Writing – original draft: JS.
13. Writing – review & editing: JS, II, VHK, IO.

## Declaration of competing interest

IO reports the following disclosures: Roche (Institutional Grant and Speakers Bureau), AstraZeneca (Advisory Board and Speakers Bureau), MSD (Advisory Board), BMS (Advisory Board), Medtronic (Institutional Grant), Intuitive (Proctorship). All other authors state that they have no conflict of interest.

## Appendix A Supplementary data

Video A Thoracic CT scan soft tissue. Video A

Video B Thoracic CT scan lung. Video B

Video C Initial gastroscopy. Video C

Video D Initial bronchoscopy. Video D

Video E Follow up bronchoscopy. Video E

## References

[1] Burt M., Diehl W., Martini N., Bains M.S., Ginsberg R.J., McCormack P.M., Rusch V.W. (1991). Malignant esophagorespiratory fistula: management options and survival. Ann. Thorac. Surg..

[2] Kim H., Khemasuwan D., Diaz-Mendoza J., Mehta A.C. (2020). Management of tracheo-oesophageal fistula in adults. Eur. Respir. Rev..

[3] Reed M.F., Mathisen D.J. (2003). Tracheoesophageal fistula. Chest Surg. Clin. N. Am..

[4] Shamji F.M., Inculet R. (2018). Management of malignant tracheoesophageal fistula. Thorac. Surg. Clin..

[5] Agha R.A., Franchi T., Sohrabi C., Mathew G., for the SCARE Group (2020). The SCARE 2020 guideline: updating consensus Surgical CAse REport (SCARE) guidelines. Int. J. Surg..

[6] Dindo D., Demartines N., Clavien P.A. (2004). Classification of surgical complications. Ann. Surg..

[7] Evens A.M., Choquet S., Kroll-Desrosiers A.R., Jagadeesh D., Smith S.M., Morschhauser F. (2013). Primary CNS posttransplant lymphoproliferative disease (PTLD): an international report of 84 cases in the modern era. Am. J. Transplant..

[8] Herth F.J., Peter S., Baty F., Eberhardt R., Leuppi J.D., Chhajed P.N. (2010). Combined airway and oesophageal stenting in malignant airway-oesophageal fistulas: a prospective study. Eur. Respir. J..

[9] Yamada S., Takai Y., Ogawa Y., Kakuto Y., Sakamoto K. (1989). Radiotherapy for malignant fistula to other tract. Cancer.

[10] Balazs A., Kupcsulik P.K., Galambos Z. (2008). Esophagorespiratory fistulas of tumorous origin. Non-operative management of 264 cases in a 20-year period. Eur. J. Cardiothorac. Surg..

[11] Lolley D.M., Ray J.F., Ransdell H.T., Razzuk M.A., Urschel H.C. (1978). Management of malignant esophagorespiratory fistula. Ann. Thorac. Surg..

[12] Grillo H.C. (1970 Jul). Surgery of the trachea. Curr. Probl. Surg..

